# Decreased Resting-State Functional Complexity in Elderly with Subjective Cognitive Decline

**DOI:** 10.3390/e23121591

**Published:** 2021-11-27

**Authors:** Huangjing Ni, Zijie Song, Lei Liang, Qiaowen Xing, Jiaolong Qin, Xiaochuan Wu

**Affiliations:** 1 School of Geographic and Biologic Information, Nanjing University of Posts and Telecommunications, Nanjing 210003, China; nihuangjing@njupt.edu.cn (H.N.); b18080404@njupt.edu.cn (Z.S.); b18080524@njupt.edu.cn (L.L.); b18080402@njupt.edu.cn (Q.X.); 2 Smart Health Big Data Analysis and Location Services Engineering Lab of Jiangsu Province, Nanjing University of Posts and Telecommunications, Nanjing 210003, China; 3 Key Laboratory of Intelligent Perception and Systems for High-Dimensional Information of Ministry of Education, School of Computer Science and Engineering, Nanjing University of Science and Technology, Nanjing 210094, China; 4 School of Science, China Pharmaceutical University, Nanjing 211198, China

**Keywords:** complexity, amplitude-aware permutation entropy, brain dynamics, short time series analysis, subjective cognitive decline

## Abstract

Individuals with subjective cognitive decline (SCD) are at high risk of developing preclinical or clinical state of Alzheimer’s disease (AD). Resting state functional magnetic resonance imaging, which can indirectly reflect neuron activities by measuring the blood-oxygen-level-dependent (BOLD) signals, is promising in the early detection of SCD. This study aimed to explore whether the nonlinear complexity of BOLD signals can describe the subtle differences between SCD and normal aging, and uncover the underlying neuropsychological implications of these differences. In particular, we introduce amplitude-aware permutation entropy (AAPE) as the novel measure of brain entropy to characterize the complexity in BOLD signals in each brain region of the Brainnetome atlas. Our results demonstrate that AAPE can reflect the subtle differences between both groups, and the SCD group presented significantly decreased complexities in subregions of the superior temporal gyrus, the inferior parietal lobule, the postcentral gyrus, and the insular gyrus. Moreover, the results further reveal that lower complexity in SCD may correspond to poorer cognitive performance or even subtle cognitive impairment. Our findings demonstrated the effectiveness and sensitiveness of the novel brain entropy measured by AAPE, which may serve as the potential neuroimaging marker for exploring the subtle changes in SCD.

## 1. Introduction

Alzheimer’s disease (AD) is a worldwide common neurodegenerative disease and has no effective cure so far. Early detection and timely intervention of AD is crucial for slowing or even stopping the progressive cognitive decline [1]. The stage of subjective cognitive decline (SCD), which is believed to precede mild cognitive impairment by years, is increasingly deemed as the earliest symptomatic manifestation of AD and at high risk for progression to preclinical or clinical state of AD [1,2]. Investigating populations with SCD is important for insight into understanding the early pathological mechanisms of AD and identifying SCD-related biomarkers [3].

SCD is characterized by accumulating amyloid pathology and neurodegeneration accompanied by very subtle cognitive decline but still within the normal cognitive performance range [4]. Currently, the diagnosis of SCD is largely based on the self-perception of worsening of memory or other cognitive functions [5]. Due to lacking objective and sensitive markers, symptoms at the SCD stage are easily dismissed as normal consequences of aging. It is hence challenging to distinguish SCD from normal aging (NA). Recently, accumulating evidence has demonstrated the promising resting-state functional magnetic resonance imaging (rs-fMRI) in revealing brain functional alterations related to the symptoms of SCD [6]. Prevailing investigations on studying SCD with rs-fMRI data are mainly focused on functional connectivity analysis [7,8,9], which highlights the interactions or temporal coherence between the blood-oxygen-level-dependent (BOLD) signals extracted from spatially separated brain regions. On the other hand, the BOLD signals per se also contain important information, especially the nonlinear dynamic characteristics [10], which can benefit to capturing the subtle changes in SCD. To date, very limited studies have been performed on the BOLD signals and mainly focused on using conventional linear statistics such as the amplitude of low-frequency fluctuation (ALFF) evaluation [11,12]. Sun et al. [11] reported the SCD group showed greater low-frequency signal amplitude than the NA group in the superior temporal, cerebellar, occipital, and inferior parietal cortex. Besides, compared to NA, Yang et al. [12] found lower amplitude and fractional ALFF in precuneus, anterior cingulum, and cerebellum in SCD. These studies have demonstrated the effectiveness of time series analysis based on BOLD signals of rs-fMRI in studying SCD. However, it remains largely unclear whether nonlinear time series analysis techniques based on BOLD signals can effectively capture the subtle changes in SCD, although the disease-induced alterations, particularly at the early stage of AD, might be better characterized by nonlinear measures [10,13].

Previous studies have demonstrated that the human brain can be deemed as a dynamic functional system presenting ongoing fluctuations in activity [14], and the range of dynamics links to the information processing capacity, which can be approximately quantified by entropy [15]. Recently, the concept of brain entropy has been adopted to quantify the state of the temporal brain dynamics. As a well-defined physical and statistical concept, brain entropy can reflect the degree of underlying randomness in a dynamic process of a time series [16]. Higher entropy indicates a stronger information processing capacity and more irregularities in brain activity, while lower entropy is linked to a higher level of predictability and impairment of brain function [16,17]. Therefore, measuring brain entropy might then provide an informative tool to assess brain functions as well as its alterations in disease [15,16,17]. In brain entropy calculation, approximate entropy and sample entropy are commonly used [15,16,18]. Nevertheless, it has been indicated that both approximate entropy and sample entropy are based solely on amplitude and ignore the temporal order of the elements in a time series [19]. Comparatively, as a symbolic dynamic measure based on the natural ordinal pattern of a time series, permutation entropy (PE) showed better performance than sample entropy in epileptic seizure prediction [20]. A more recent rs-fMRI research on AD also described the novel application of PE in brain entropy calculation and found decreased complexity in AD [17]. Nevertheless, PE only considers the sample order and ignores the amplitude information in the time series, which might have a detrimental effect on the performance [21]. Recently, Azami et al. developed the amplitude-aware PE (AAPE) algorithm, which is sensitive to variations in the amplitude and the frequency of the time series while considering the ordinal patterns [22]. It has been demonstrated that compared with PE, AAPE is more flexible in the quantification of the ordinal patterns and more powerful in detecting subtle changes in the time series [21].

The primary goal of this study is to introduce AAPE as a novel measure of brain entropy to explore the group differences between NA and SCD. Therefore, we hypothesized that the subtle alterations in SCD could be reflected by the complexity information contained in BOLD signals, and the complexity can be detected by AAPE. Meanwhile, the complexity of BOLD signals should be reduced in the SCD group, compared with the NA group. Additionally, we will further explain the implications of the altered complexity by relating AAPE to the neuropsychological measurements.

## 2. Materials and Methods

### 2.1. ADNI Study Design

Data used in the article were obtained via the Alzheimer’s Disease Neuroimaging Initiative (ADNI) database (http://adni.loni.usc.edu accessed on 16 February 2021). The ADNI was initially launched in 2003 (ADNI-1), and additional recruitment was made through ADNI-GO in 2009, ADNI-2 in 2010, and ADNI-3 in 2016. The primary goal of the ADNI has been to identify serial MRI, PET, other biological markers, and clinical and neuropsychological assessments that would support the early detection and tracking of AD, and improve clinical trial design. The ADNI study has observed individuals diagnosed as cognitively normal or with varying degrees of cognitive impairment since 2005. The ADNI battery includes broad neuroimaging and clinical and neuropsychological assessments. For up-to-date information, see http://www.adni-info.org (accessed on 21 October 2021). The study subjects were recruited from over 50 sites across the US and Canada. They gave the written informed consent at the time of enrolment for imaging and genetic sample collection and completed the questionnaires approved by the Institutional Review Board (IRB) of each participating site.

### 2.2. Participants

In the ADNI database, participants labeled as SMC are recruited into a significant memory concern cohort. In this study, we included these participants as the SCD group. The common inclusion criteria for both NA and SCD were as follows: (i) having a Mini-Mental State Examination (MMSE) score between 24 and 30; (ii) having a clinical dementia rating (CDR) score of 0 and the memory box score of 0; (iii) having a normal Wechsler Memory Scale Logical Memory II subscale (in detail: ≥9 for subjects with 16 or more years of education; ≥5 for subjects with 8~15 years of education; and ≥3 for 0~7 years of education); (iv) having a Geriatric Depression Scale (GDS) score less than 6; and (v) having no objective cognitive impairments in memory or activities of daily living. Additionally, the NA participants must be free of memory complaints and would be verified by a study partner. On the contrary, the SCD participants must have a significant subjective memory concern as reported by himself/herself, study partner or clinician and confirmed by using the Cognitive Change Index (CCI) questionnaire with the total score from the first 12 items ≥ 16 [23].

After discarding the participants with either corrupted functional images or structural images and only choosing one scan for each participant, we finally included 43 SCD individuals and 43 well-matched NAs in this study. All participants underwent structural scans, rs-fMRI scans, and comprehensive neuropsychological assessments. The rs-fMRI scanning parameters: TR/TE as 3000/30 ms and flip angle (FA) of 90°. Each series had 197 volumes, and each volume consisted of 48 image slices with dimensions 64 × 64 and voxel size 3.44 × 3.44 × 3.4 mm^3^. Sagittal structural images with a resolution of 1 × 1 × 1.2 mm^3^ were acquired using a magnetization prepared rapid gradient echo (MPRAGE) 3D T1-weighted sequence (TR = 6.8 ms; TE = 3.16 ms; FA = 9°). Table 1 summarized the demographic, clinical, and neuropsychological information of the participants involved in this study.

### 2.3. Neuropsychological Assessments

All participants underwent the neuropsychological assessments involving the global cognitive function measures of CDR [24] and MMSE [25], as well as the evaluations of the Preclinical Alzheimer’s Cognitive Composite (PACC) [26,27]. The CDR describes six categories of cognitive functioning including memory, orientation, judgment and problem solving, community affairs, home and hobbies, and personal care. The total score of these six categories (CDRSB) was used in this study as the global severity of dementia with higher scores indicative of the presence of dementia. Moreover, by mainly evaluating orientation, memory, attention, concentration, naming, repetition, and comprehension, the MMSE is scored as the number of correctly completed items with lower scores indicative of poorer performance and greater cognitive impairment. In addition, the PACC was designed to serve as the primary outcome measure for trials conducted at the asymptomatic phase of AD [26,27]. In ADNI, the modified PACC assessment captures performance on tasks of episodic memory, orientation, and executive function, which are the prominent domains of AD-related cognitive dysfunction. Specifically, the Digit Symbol Substitution Test (DSST) was used to calculate the PACC-DSST score, while the PACC-LogTMTB score employed the log-transformed completion time of the Trail Making Test B (TMTB) [28]. The raw component scores of PACC-DSST and PACC-LogTMTB were z-score standardized, and greater scores reflected better cognitive performance. The information of CDRSB, MMSE, PACC-DSST, and PACC-LogTMTB for both NA and SCD groups were summarized in Table 1.

### 2.4. Data Preprocessing

**Figure 1:**
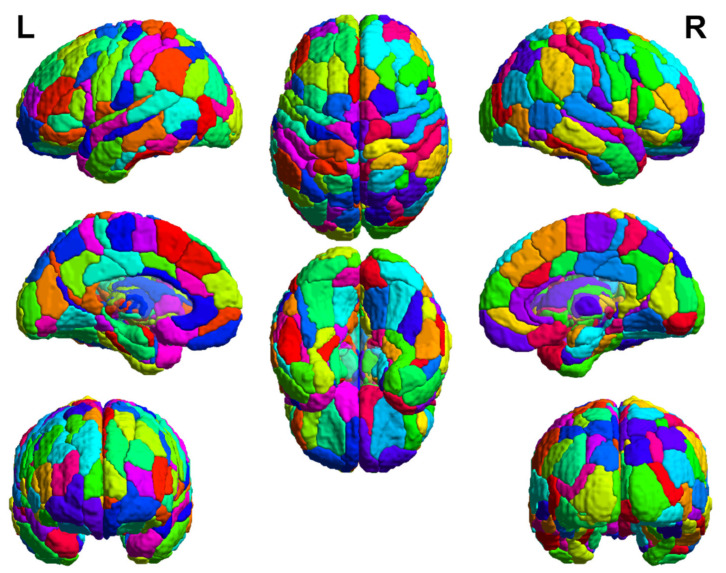
Illustrations of the Brainnetome atlas in the cerebral regions. These 3D brains with multiple views are rendered in standard MNI space, and different colors denote different subregions. L and R represent left and right hemispheres, respectively.

All functional imaging data preprocessing was performed using the Data Processing Assistant for Resting-State fMRI Advanced Edition [29,30], which is based on Statistical Parametric Mapping (SPM12) [31]. The first seven volumes were discarded and the remaining 190 volumes were first corrected for timing differences and motion effects. None of the participants were excluded on the criterion of more than 3 mm of translation or 3° of rotation in any direction. Next, the nuisance covariates including linear and quadratic trend, Friston 24 motion parameters, white matter signal, and cerebral spinal fluid signal were regressed. In order to remove the spiking influence caused by motion artifacts, a despiking step was adopted [32]. Afterward, the functional brain images were normalized to the Montreal Neurological Institute (MNI) space using T1 image unified segmentation and resampled to voxel size of 2 × 2 × 2 mm^3^. Finally, spatially smoothed with a 4 mm isotropic Gaussian kernel was conducted. In order to calculate the AAPE values at the regions of interests (ROI) level, the time series of each voxel in the cerebral regions of the Brainnetome atlas [33] were firstly extracted for AAPE analysis. The subsequent regional AAPE values in 210 cortical and 36 subcortical ROIs in the Brainnetome atlas were obtained by averaging the AAPE values of all voxels within each ROI. An illustration of the Brainnetome atlas is shown in Figure 1, and more details of the Brainnetome atlas can be found via http://atlas.brainnetome.org/download.html (accessed on 21 October 2021).

### 2.5. AAPE Algorithm

In this study, the AAPE algorithm [22] is employed for complexity analysis of the time series. Specifically, let us consider a time series $X=\{x_{1},x_{2},\cdots ,x_{N}\}$ with length *N* and an embedded dimension *m*. The first step is to form *N* − *m* + 1 vectors of size *m* samples, such that $X_{m}(i)=\{x_{i},x_{i+1},\cdots ,x_{i+m-1}\}$ (1 ≤ *i* ≤ *N* − *m* + 1). When $X_{m}(i)$ is re-arranged in ascending order, a new order vector defined as an ordinal pattern emerges as $\pi_{i}^{m}=\{\pi_{0},\pi_{1},\cdots \pi_{m-1}\}$ such that $x_{i+\pi_{0}}\leq x_{i+\pi_{1}},\cdots ,\leq x_{i+\pi_{m-1}}$. Thus, there are potentially *m*! different ordinal patterns, termed $\Pi_{j}^{m}$ (0 ≤ *j* ≤ *m*!), can be obtained from vectors $X_{m}$. Next, the appearance probability of each ordinal pattern $\pi_{k}$, termed as $p(\pi_{k})$, can be estimated. In order to calculate the $p(\pi_{k})$ for each $\pi_{k}$, the average absolute (*AA*) and relative amplitudes (*RA*) of vectors $X_{m}$ should be firstly calculated out. These amplitudes for a specific vector $X_{m}(i)$ can be obtained as

(1) $$AA_{i}=\frac{1}{m}\sum_{l=1}^{m}\left|x(i+l-1)\right|,$$

and

(2) $$RA_{i}=\frac{1}{m-1}\sum_{l=2}^{m}\left|x(i+l-1)-x(i+l-2)\right|$$

Next, the relative frequency of $\pi_{k}$ can be computed as

(3) $$p(\pi_{k})=\frac{\sum_{i=1}^{N-m+1}\delta (\pi_{k},\Pi^{m})\cdot (K\cdot AA_{i}+(1-K)\cdot RA_{i})}{\sum_{i=1}^{N-m+1}K\cdot AA_{i}+(1-K)\cdot RA_{i}}$$

where δ(u,v) is the Kronecker delta function modified to work with the ordinal patterns, i.e.,

(4) $$\delta (u,v)=\left\{\begin{array}{ll} 1, & \;if\;u(i)=v(i),\;\;\forall i=1,2,\cdots ,m\\ 0, & \;otherwise \end{array}\right.$$

and *K* is an adjusting coefficient related to the terms *AA* and *RA*, ranging from 0 to 1. Finally, the AAPE value can be computed as

(5) $$AAPE(m)=-\sum_{k=1}^{m!}p(\pi_{k})\cdot \ln(p(\pi_{k}))$$

As for the case of $p(\pi_{k})=0$, it is specified that 0⋅ln0=0. AAPE can characterize the local order structure of the time series. The higher the diversity of ordinal patterns in a time series is, the larger the value of AAPE is. Therefore, a large AAPE value indicates a more random time series, whereas a small AAPE value indicates that the time series is regular.

In the process of AAPE calculation, only two parameters (i.e., *m* and *K*) are required to be set. A proper selection of *m* is key to obtaining robust entropy estimation. The higher is *m*, the more reliable is the value of AAPE [34]. Additionally, Amigó et al. [35] suggested *N* > 5*m*! should be satisfied, and in practice, Bandt et al. [19] recommended a range for *m* should between 3 and 7. As the total length of BOLD signals is 190, the appropriate *m* can thus be set as 3 or 4. Nevertheless, in the current study, the subtle group differences between NA and SCD can only be characterized in the case of *m* = 4. In addition, for the parameter *K*, it is the adjusting coefficient to set the importance of the average of amplitudes (i.e., *AA*) and the differences of the amplitude values (i.e., *RA*). If *K* < 0.5, it means that *RA* is much more important, while *K* > 0.5 puts more emphasis on *AA*. In contrast, if both *AA* and *RA* are equally important, the setting *K* = 0.5 is recommended [22]. After trying different *K* values (e.g., *K* = 0.1, 0.5, and 0.9), we found that AAPE is not very sensitive to the values of *K* in the context of BOLD signal analysis. Therefore, we adopt *m* = 4 and *K* = 0.5 in this work.

### 2.6. Statistical Analysis

We analyzed the demographic, cognitive, and neuropsychological data using the two-tailed chi-square test for categorical data and two-tailed two-sample *t*-test for continuous data. Then, we examined the AAPE values between NA and SCD groups at the ROI level. In detail, we firstly performed a two-sample *t*-test for each ROI. The subsequent multiple comparisons were corrected by using a false discovery rate (FDR) [36] for identifying those ROIs with statistically significant differences. Furthermore, to investigate the relationships between the AAPE and the neuropsychological assessments, a standard nonparametric regression analysis [37] was firstly conducted in the SCD group within the ROIs showing significant group differences in AAPE, with their age, sex, years of education, mean frame-wise displacement (FD), ADAS13, ADAS-Word and GDS as covariates. Moreover, the nonparametric regression and statistical inference was performed on the statistical library for least squares support vector machines (StatLSSVM) toolbox (http://www.esat.kuleuven.be/stadius/statlssvm/ (accessed on 21 October 2021)). To improve interpretability in regression models, we adopted the simple linear kernel while tunning the smoothing parameters using 10-folder cross-validation criterion. Subsequently, based on the trained regression model, we further predicted the scores of neuropsychological assessments by employing the AAPE values. The Pearson correlations coefficients between the ground truth and predicted neuropsychological scores were thus calculated to examine the effect of the regression model. Bonferroni correction was carried out to adjust multiple comparisons.

## 3. Results

### 3.1. Complexity Differences of ROIs between NA and SCD Groups

**Figure 2:**
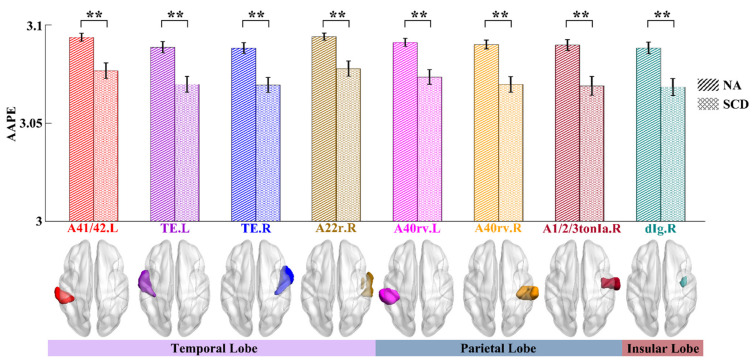
Results of intergroup differences in complexity between NA and SCD groups. In the upper panel, the bar chart illustrates group-averaged AAPE values of the eight ROIs with significant differences between NA (right-hatched bars) and SCD (cross-hatched bars) groups. The abscissa is the ROI names in Brainnetome atlas, and the ordinate reflects their AAPE values. Compared with the NA group, the mean AAPE values of the SCD group are slightly lower. The error bars indicate corresponding standard errors. Statistical significance: ** *p* < 0.01 (FDR corrected). Different colors for the bar chart and ROI names correspond to their same color-coded 3D brain shown in the axial view (middle panel). In the lower panel, the bands colored by the light purple, the gray-blue and the light red indicate the temporal lobe, the parietal lobe and the insular lobe, respectively. Their range of length covers the corresponding ROIs in the upper and middle panels.

We firstly obtained the regional AAPE as the complexity by averaging the AAPE values of all voxels within each ROI in the Brainnetome atlas. Next, by performing the two-sample *t*-test on each ROI, eight ROIs can be found to exhibit significant differences (*p* < 0.01, FDR corrected) between NA and SCD groups. As shown in Figure 2, the average AAPE values of the SCD group were relatively lower than those of the NA group for all of the eight ROIs. These ROIs included the subregions of the bilateral superior temporal gyrus (STG) (A41/42.L, TE.L, TE.R and A22r.R), the rostroventral areas of the bilateral inferior parietal lobule (IPL) (A40rv.L and A40rv.R), the tongue and larynx region of the right postcentral gyrus (PoG) (A1/2/3tonIa.R) and the dorsal granular insula of the right insular gyrus (INS) (dIg.R). They were predominantly in the temporal and parietal lobes as well as the insular lobe.

### 3.2. Relationships between AAPE and Neuropsychological Assessments

In the SCD group, we performed the standard nonparametric regression analysis to model the potential relationships between AAPE values in these eight ROIs and neuropsychological assessments, while controlling for age, sex, education, mean FD, ADAS13, ADAS-Word, and GDS. For the CDRSB scores, we observed the linear decline trend presented in the A41/42.L, the A40rv.L, and the A40rv.R. For the MMSE scores, the obviously upward trend can be found in the TE.R and dIg.R. No other trends revealed by the standard nonparametric regression were observed for either PACC-DSST or PACC-LogTMTB.

Furthermore, based on the trained regression model, we can predict the scores of neuropsychological assessments by using 10-folder cross validation and correlated them with the real scores. As shown in Table 2, it is exhibited that good predictions for the CDRSB scores can be obtained in the A41/42.L (*r* = 0.39, *p* = 0.0099), the A40rv.L (*r* = 0.49, *p* = 0.0009), and the A40rv.R (*r* = 0.43, *p* = 0.0043), while good predictions for the MMSE scores were observed in the TE.R (*r* = 0.34, *p* = 0.028) and the dIg.R (*r* = 0.4, *p* = 0.0075).

**Figure 3:**
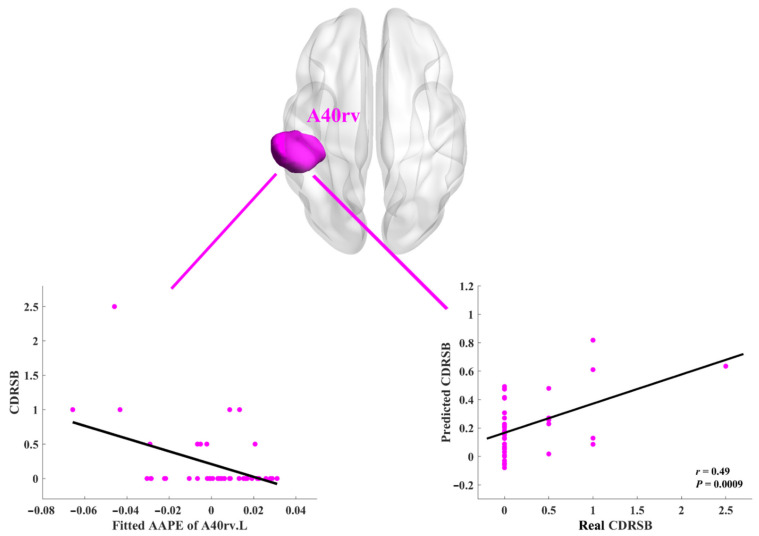
Results of clearly decreased trend and good predictions between the A40rv.L and CDRSB after Bonferroni correction for multiple ROIs and multiple measures (*p* < 0.05/32). The involved ROI is rendered onto the 3D brain shown in the axial view, and colored with magenta (the upper panel). The ROI name is also shown in magenta. In the left bottom panel, the abscissa represents the residuals of AAPE values for all the SCD participants in A40rv.L after removing the covariates of age, sex, education, meanFD, ADAS13, ADAS-Word, and GDS, whereas the ordinate represents the corresponding CDRSB scores for each SCD participant. The black solid line clearly presented the trend of data distribution, which was calculated by the standard nonparametric regression. In the right bottom panel, the abscissa and ordinate represent the real and predicted CDRSB scores respectively, and the black solid line is the fitting line. The values of *r* is the Pearson correlation coefficients, while *p* values indicate the level of statistical significance.

Additionally, after Bonferroni correction for multiple ROIs and multiple measures, we only found the clearly decreased trend and good prediction in the A40rv.L and the measure of CDRSB. The results were presented in Figure 3. Generally, the AAPE values in the subregions of STG, IPL, and INS were related to the neuropsychological assessments of CDRSB and MMSE.

## 4. Discussion

In this study, we introduced AAPE as the novel measure of brain entropy and adopted it to explore the group differences in complexity between NA and SCD. The significant differences were mainly distributed in the temporal, parietal, and insular lobes. At the ROI level, the SCD group exhibited significantly lower AAPE values (lower complexities) in eight ROIs, involving the subregions of STG, IPL, PoG, and INS. Furthermore, we conducted the standard nonparametric regression analysis between AAPE values of these eight ROIs and the neuropsychological assessments. For CDRSB, we observed the linearly declined trend in the left A41/42 and the bilateral A40rv, while for MMSE, the clear upward trend was found in the TE.R and dIg.R. To summarize, we found significantly decreased complexity in the SCD group, and the lower complexity may indicate poorer cognitive performance or more likely to present subtle cognitive impairment.

As defined by the SCD-initiative, the SCD criteria include two major features: (i) a self-experienced persistent decline in cognitive capacity and unrelated to an acute event; and (ii) normal performance on standardized cognitive tests used to classify mild cognitive impairment [2]. Thereby, the diagnosis of SCD is largely dependent on self-reported cognitive decline or memory issues. Nevertheless, such subjective feelings are easy to be ignored by patients, which might lead to missed diagnosis [38]. In this case, there is an urgent need to find objective biomarkers for identification of individuals at increased risk of SCD. On the other hand, the cognitive performance of SCD usually shows no evidence of objective cognitive impairment by neuropsychological testing and in daily functioning [39]. It might be the reason why significant group differences were not observed between NA and SCD in this study by using the assessments of CDRSB, MMSE, ADAS13, FAQ, GDS, PACC-DSST, and PACC-LogTMTB (Table 1). From the results, it may suggest the inadequate sensitiveness of the commonly used clinical and neuropsychological assessments in identifying SCD [40]. In contrast, the subtle and substantial alterations in SCD can be captured by AAPE, which indicates the superiority of AAPE. As an objective and sensitive measure, AAPE can characterize the implicit irregularity of non-stationary BOLD signals and effectively explore the significant ROIs with group differences between NA and SCD, which might provide useful features for identification of individuals with SCD. Therefore, our results suggest that the novel brain entropy measured by AAPE might be served as the potential neuroimaging marker for identifying SCD.

Two potential reasons could contribute to the outstanding results of AAPE. First, brain entropy analysis is particularly suited to characterize the intrinsic abnormality in SCD. The reserved normal cognitive performance in SCD is related to the adaptive capacity of the human brain [41]. As indicated in [42], normal physiology requires an intricate network to control function effectively, and greater underlying system complexity better enables a system to restore the steady-state after perturbation. With aging and disease, there is a loss of complexity in the dynamics of the brain, and hence optimal performance may not be maintained [43]. Thus, complexity is a suitable measure of adaptive capacity, which can reflect the ability of the brain to adapt to the changing internal and external functional activities. In the studies of brain aging and disease, brain entropy is a well-established measure of complexity. Therefore, exploring brain entropy could be effective to measure the complexity in BOLD signals and be a useful marker of aging and disease-related decline. Second, the advantages of the AAPE algorithm benefit to improve the sensitiveness of the subtle changes detection in the BOLD signals of SCD. The introduced AAPE algorithm is an improvement of PE. On the one hand, AAPE considers the relative order relations of neighboring values in the time series, which is robust to the noisy BOLD signal. On the other hand, AAPE incorporates the amplitude information into the ordinal patterns, which could lead to more precise change detection in the BOLD fluctuation and more accurate brain entropy calculation. A recent study from Cuesta [21] performed a systematic comparative analysis and confirmed that the classification performances of using both ordinal patterns and amplitude information simultaneously outperform those of using ordinal patterns or amplitude isolatedly. Therefore, the introduced AAPE as the measure of brain entropy could explore the group differences between NA and SCD effectively and sensitively.

Our investigation identified eight ROIs that exhibit significant differences between NA and SCD groups. These ROIs, located in the temporal, parietal, and insular lobes, are from both brain hemispheres and mainly cover part of STG, IPL, PoG, and INS. They are consistent with previous studies that used conventional linear measures for SCD detection. For example, as indicated in [11,44], STG and IPL were reported to show abnormal in SCD by using ALFF values. Li et al. assessed the intrinsic connectivity network of SCD individuals and found that SCD individuals show lower degree centrality in the IPL than NAs [8]. Kim et al. found the PoG altered in SCD by conducting network-based statistics analysis [45]. By conducting functional connectivity analysis, Xu et al. [46] found the decreased functional connections in SCD were mainly distributed in the temporal lobe, thalamus, and INS. Besides, SCD individuals had more severe topological network impairment and a higher pathological burden than NAs, especially in the temporal and parietal lobes [47], which is also in line with our findings (see Figure 2). Moreover, benefitting from the Brainnetome atlas, we can obtain finer functional subregions with significant intergroup differences than before. For example, in the STG, we can further identify the abnormal subregions in the left area 41/42 (i.e., A41/42.L), the left TE1.0 (i.e., TE.L), the right TE1.2 (i.e., TE.R) and the right rostral area 22 (i.e., A22r.R). Likewise, in the IPL, the finer abnormal subregions can be found in the bilateral rostroventral area 40 (i.e., A40rv.L and A40rv.R). The finer results can provide a more accurate target of functional abnormal in SCD individuals, which may be valuable for precision treatment. Generally, these previous studies support that our identified ROIs are reasonable and closely related to SCD.

Additionally, it is worthy to further explore why these significant ROIs show reduced functional complexity in the SCD group. Let us now turn to the BOLD signals that derived the time series, on which the AAPE calculation is based. The BOLD signal emerges from changes in the deoxyhemoglobin concentration in cerebral venous circulation, indirectly reflecting a feedback hemodynamic adaptation to cerebral metabolic activity [48]. Previous studies have found significantly hypometabolic in SCD, such as in the STG and the IPL [49,50]. It is proposed that hypometabolism probably began to emerge in certain brain regions and finally spread over the entire AD-related metabolic pattern with gradual destruction of the neurons [3,51]. Based on this, we speculate that individuals with SCD emerge regional metabolically impaired changes, which might indicate local processing inefficiencies in the observed ROIs. Hence, it may be a reason that leads to the declination of functional complexity.

In addition, it is also important to explore the neuropsychological meanings of the complexity changes measured by AAPE. In all the eight ROIs with significant intergroup differences, the AAPE values of the NA group are generally larger than those of the SCD group. As indicated above, larger AAPE values indicate more irregular time series and correspond to higher complexity. As shown in Figure 3, in the A40rv.L, a clearly decreased trend can be observed between AAPE and CDRSB scores in SCD participants. Moreover, the CDRSB score can also be well predicted using the trained model (*r* = 0.49, *p* < 0.0016, Bonferroni corrected). Apart from this, the AAPE values from the TE.R and dIg.R present a clear upward trend. The neuropsychological assessments of CDRSB and MMSE are comprehensive indicators that can measure various categories of cognitive functions [24,25], mainly including episodic memory, orientation, and executive function. These three key cognitive categories commonly require to be evaluated in preclinical AD [27]. Specifically, the MMSE score is effective for cognitive screening of the elderly and lower MMSE scores indicate greater cognitive impairment. Similarly, the CDRSB score is a frequently used index of cognitive decline. Larger CDRSB scores can reflect poorer cognitive performance. Consequently, it can be inferred that lower complexity in SCD may correspond to poorer cognitive performance or possible subtle cognitive impairment.

There exist several limitations in the current study. First, the relatively small number of participants limits our current research, which may reduce the statistical power. The total number of participants including NA and SCD is eighty-six, which is the currently common sample size in SCD studies [6]. Although the subtle differences between NA and SCD groups have been detected in the current research, the relatively small group size could limit the interpretation of our results. Therefore, a validation of our results on larger SCD datasets should be further conducted. Second, the length of the rs-fMRI time series used for AAPE analysis in the current study is relatively short, although this is a typical configuration in the widely used ADNI dataset. One of the advantages of the introduced AAPE algorithm is its robustness to short signals [22]. Nevertheless, larger values of the embedded dimension could contain more ordinal patterns, which is helpful to explore the intrinsic complex dynamics characteristics [21]. In this study, in the case of the embedded dimension being *m* = 3, only *m*! = 6 ordinal patterns can be derived, which is insufficient to characterize the subtle group differences between NA and SCD. By contrast, the 24 ordinal patterns derived from *m* = 4 are more powerful. Further investigations on longer time series will be our future work, which may better evaluate the abnormal complexity of BOLD signals in SCD. Third, the intergroup differences were obtained from a cross-sectional design used in the research, which might be influenced by the individual differences among the employed participants. Considering the reported heterogeneity in NA and SCD individuals [52], further longitudinal studies may be more appropriate to closely confirm the findings and will be explored in the future. Moreover, it could be interesting to conduct a systematic study to explore the performances of different measures at the individual level, such as the Hurst exponent and multifractal measures, the commonly used ALFF evaluation, as well as the classic entropy measures.

## 5. Conclusions

This study introduced AAPE as the novel measure of brain entropy to investigate the intergroup differences in complexity between NA and SCD. We found significant group differences presented in the subregions of STG, IPL, PoG, and INS. Furthermore, we also found decreased complexity in the SCD group and found that the complexities in all the NA and SCD participants were significantly correlated with neuropsychological assessments (i.e., the CDRSB, MMSE, and PACC-LogTMTB). The lower AAPE values in SCD may indicate poorer cognitive performance or possible subtle cognitive impairment. These findings demonstrate that the subtle changes in SCD can be effectively and sensitively explored by the novel brain entropy, AAPE, which may serve as an assistant and potential neuroimaging marker for the detection of SCD.

## Figures and Tables

**Table 1:** The demographic, clinical, and neuropsychological information of the participants involved in this study.

	NA	SCD	*p* Value
Number of participants	43	43	-
Years of age	73.57 ± 3.27	75.48 ± 5.66	0.0584 ^a,c^
Sex (Male/Female)	17/26	14/29	0.5005 ^b,c^
Years of education	16.33 ± 2.35	16.37 ± 2.90	0.9351 ^a,c^
CDRSB	0.15 ± 0.55	0.21 ± 0.48	0.6029 ^a,c^
MMSE	28.98 ± 1.14	29.14 ± 0.97	0.4778 ^a,c^
ADAS13	9.26 ± 5.32	7.91 ± 4.58	0.2138 ^a,c^
ADAS-Word	2.88 ± 1.94	2.00 ± 1.48	0.0199 ^a,d^
FAQ	0.23 ± 0.84	0.48 ± 0.89	0.1980 ^a,c^
GDS	1.12 ± 1.89	1.30 ± 1.12	0.5809 ^a,c^
CCI	-	24.91 ± 2.06	-
PACC-DSST	−0.18 ± 4.06	0.53 ± 2.90	0.3531 ^a,c^
PACC-LogTMTB	−0.14 ± 3.64	0.34 ± 2.74	0.4909 ^a,c^

Abbreviation: NA, normal aging; SCD, subjective cognitive decline; CDRSB, Clinical Dementia Rating sum of boxes score; MMSE, Mini-Mental State Examination; ADAS13, 13-item Alzheimer’s Disease Assessment Scale; ADAS-Word, the item of Delayed Word Recall test extracted from ADAS13; FAQ, Functional Assessment Questionnaire; GDS, Geriatric Depression Scale; CCI, Cognitive Change Index; PACC, Preclinical Alzheimer’s Cognitive Composite; DSST, Digit Symbol Substitution Test; LogTMTB, Log Tansformed Trail Making Test B; plus-minus score values are mean±std. The symbol “-” denotes no calculation or absence of records. ^a^ The *p* value was obtained by two-sample two-tailed *t* test. ^b^ The *p* value was obtained by two-tailed chi-square test. ^c^ The *p* value with *p* > 0.05 indicated no statistically significant differences between the two groups. ^d^ The *p* value with *p* < 0.05 indicated there existed statistically significant differences between the two groups.

**Table 2:** Results of Pearson correlation analysis between the neuropsychological assessments of ground truth and predicted scores.

Gyrus	ROI	CDRSB (*r*, *p*)	MMSE (*r*, *p*)	PACC-DSST (*r*, *p*)	PACC-LogTMTB (*r*, *p*)
STG	A41/42.L	0.39, 0.0099 *	0.25, 0.1086	0.05, 0.7445	0.15, 0.3466
	TE.L	0.28, 0.0728	0.24, 0.116	0.02, 0.9041	0.01, 0.9314
	TE.R	0.29, 0.0633	0.34, 0.028 *	0.02, 0.8882	0.01, 0.9273
	A22r.R	0.20, 0.1935	0.15, 0.335	0.03, 0.8589	0.07, 0.6457
IPL	A40rv.L	0.49, 0.0009 **	0.26, 0.0878	0.08, 0.6174	0.16, 0.3178
	A40rv.R	0.43, 0.0043 *	0.1, 0.5174	0.04, 0.8164	0.08, 0.6197
PoG	A1/2/3tonIa.R	0.18, 0.261	0.2, 0.1902	0.01, 0.9342	0.05, 0.7556
INS	dIg.R	0.11, 0.5008	0.4, 0.0075 *	0.11, 0.4815	0.12, 0.4289

In the table, *r* is the Pearson correlation coefficient, and *p* indicates the level of statistical significance. * represents *p* < 0.05, while ** represents significance at Bonferroni correction for multiple ROIs *p* < 0.0016 (*p* < 0.05/32). Abbreviation: STG, Superior Temporal Gyrus; IPL, Inferior Parietal Lobule; PoG, Postcentral Gyrus; INS, Insular Gyrus; ROI, Regions of Interests; CDRSB, Clinical Dementia Rating sum of boxes score; MMSE, Mini-Mental State Examination; PACC, Preclinical Alzheimer’s Cognitive Composite; DSST, Digit Symbol Substitution Test; LogTMTB, Log-Transformed Trail Making Test B.

## Data Availability

Publicly available datasets were analyzed in this study. This data can be found here: http://www.adni-info.org/ (accessed on 21 October 2021).
